# CTB-targeted protocells enhance ability of lanthionine ketenamine analogs to induce autophagy in motor neuron-like cells

**DOI:** 10.1038/s41598-023-29437-8

**Published:** 2023-02-13

**Authors:** Maria A. Gonzalez Porras, Heather M. Gransee, Travis T. Denton, Dunxin Shen, Kevin L. Webb, C. Jeffrey Brinker, Achraf Noureddine, Gary C. Sieck, Carlos B. Mantilla

**Affiliations:** 1 Department of Anesthesiology & Perioperative Medicine, Mayo Clinic, Rochester, MN USA; 2 Department of Physiology & Biomedical Engineering, Mayo Clinic, Rochester, MN USA; 3 Department of Biomedical Engineering, University of Texas at San Antonio, San Antonio, TX USA; 4 Department of Pharmaceutical Sciences, College of Pharmacy and Pharmaceutical Sciences, Washington State University Health Sciences Spokane, Spokane, WA USA; 5 Department of Translational Medicine and Physiology, Elson S. Floyd, College of Medicine, Washington State University Health Sciences Spokane, Spokane, WA USA; 6 Steve Gleason Institute for Neuroscience, Washington State University Health Sciences Spokane, Spokane, WA USA; 7 Center for Micro-Engineered Materials, University of New Mexico, Albuquerque, NM USA; 8 Department of Chemical and Biological Engineering, University of New Mexico, Albuquerque, NM USA; 9 Department of Molecular Genetics and Microbiology, University of New Mexico, Albuquerque, NM USA; 10 MB2-758, St Mary’s Hospital, Mayo Clinic, 200 First St SW, Rochester, MN 55905 USA

**Keywords:** Nanoparticles, Autophagy, Motor neuron

## Abstract

Impaired autophagy, a cellular digestion process that eliminates proteins and damaged organelles, has been implicated in neurodegenerative diseases, including motor neuron disorders. Motor neuron targeted upregulation of autophagy may serve as a promising therapeutic approach. Lanthionine ketenamine (LK), an amino acid metabolite found in mammalian brain tissue, activates autophagy in neuronal cell lines. We hypothesized that analogs of LK can be targeted to motor neurons using nanoparticles to improve autophagy flux. Using a mouse motor neuron-like hybrid cell line (NSC-34), we tested the effect of three different LK analogs on autophagy modulation, either alone or loaded in nanoparticles. For fluorescence visualization of autophagy flux, we used a mCherry-GFP-LC3 plasmid reporter. We also evaluated protein expression changes in LC3-II/LC3-I ratio obtained by western blot, as well as presence of autophagic vacuoles per cell obtained by electron microscopy. Delivering LK analogs with targeted nanoparticles significantly enhanced autophagy flux in differentiated motor neuron-like cells compared to LK analogs alone, suggesting the need of a delivery vehicle to enhance their efficacy. In conclusion, LK analogs loaded in nanoparticles targeting motor neurons constitute a promising treatment option to induce autophagy flux, which may serve to mitigate motor neuron degeneration/loss and preserve motor function in motor neuron disease.

## Introduction

Autophagy is a catabolic process that facilitates the removal of damaged organelles and prevents accumulation of toxic proteins by the degradation of cytoplasmic components through lysosomal pathways^1,2^. Autophagy is recognized as an important process that not only responds to conditions of nutrient deprivation, but also maintains homeostasis with effects on cell metabolism, cell growth, balance between cell survival and cell death, immune surveillance, degeneration, plasticity and aging^3–5^. In the autophagy process, the targeted cellular components are sequestered by an isolation membrane (phagophore) that eventually forms a double membrane structure (autophagosome)^6,7^. The cargo gets degraded when the autophagosome fuses with the lysosome^8,9^. In motor neurons, autophagy is as a key regulator of cellular homeostasis and plasticity and plays important roles in neuromuscular junction morphology^10–13^, survival^8^, synaptic plasticity^14,15^ and axonal pruning^16^. Impaired autophagy has been implicated in neurodegenerative diseases, including motor neuron disorders^17–19^. Therefore, upregulation of autophagy is a promising therapeutic approach for motor neuron diseases and disorders. Despite great potential, there is no current intervention that specifically modulates autophagy in humans.

Lanthionine ketenamine (LK) is a sulfur amino acid metabolite found in mammalian brain tissue at low concentrations with no known natural function. LK, and its more cell membrane permeable analog lanthionine ketenamine ethyl ester (LKE), possess potent neuroprotective, neurotrophic, and anti-neuroinflammatory properties^20–26^. Both LK and LKE slow down the decline of motor functions in a mouse model of amyotrophic lateral sclerosis^27^ and activate autophagy in rat glioma and human neuroblastoma cells^28^. The need for new compounds to enhance autophagy and fight against neurodegenerative diseases has recently been addressed by the synthesis of new analogs of LK and LKE^25,29^. For instance, phosphonate analogs of LK (lanthionine ketenamine phosphonates (LK-Ps)) or LKE (lanthionine ketenamine (ester) phosphonates (LKE-Ps)) can enhance target engagement of LK^29^. We hypothesized that LK analogs can be targeted specifically to motor neurons using nanoparticles to induce autophagy flux. We previously developed a motor neuron targeting platform that delivers intracellular cargo^30,31^. This nanoparticle platform consists of a mesoporous silica nanoparticle (MSN) core encapsulated by a supported lipid bilayer (protocells^32^) and engineered to target motor neurons by modifying the lipid bilayer with the atoxic subunit B of cholera toxin B (CTB). The purpose of using liposomes and MSNs is to have the combined beneficial features of both. The MSN core was used because of the following characteristics: (a) silica has been identified as Generally Recognized As Safe (GRAS) by FDA rendering possible its use in medical applications in vivo on a wider spectrum; (b) fabrication of MSN via the sol–gel process is straightforward and controlled, allowing for independent modification of MSN morphology and the surface chemistry. This feature enhances cargo loading and protection when compared to other common drug delivery systems (e.g., liposomes); and (c) the degradation of MSNs and the associated cargo release rate can be modulated by modifications of the silica matrix properties. Encapsulation of the MSN within a supported lipid bilayer (to form protocells) permits: (a) enhanced protection of the cargo loaded in the MSNs; and (b) introduction of strategic targeting ligands into the supported lipid bilayer to target cell specifically^32^. Our targeting strategy, CTB, binds the ganglioside GM1 present in neuronal membranes^33^. By coating protocells with CTB (CTB-protocells) we have shown that they specifically target motor neurons when compared to muscle cells, engage in retrograde transport in motor neurons and can deliver small molecule cargo^30,31^. By loading CTB-protocells with LK analogs, we aim to develop an autophagy modulator that specifically targets motor neurons. The results of this study will provide valuable information for future therapeutic approaches to modulate autophagy specifically in motor neurons using a targeted delivery system.

## Results

### CTB-protocells were successfully generated and loaded with three LK analogs

**Figure 1:**
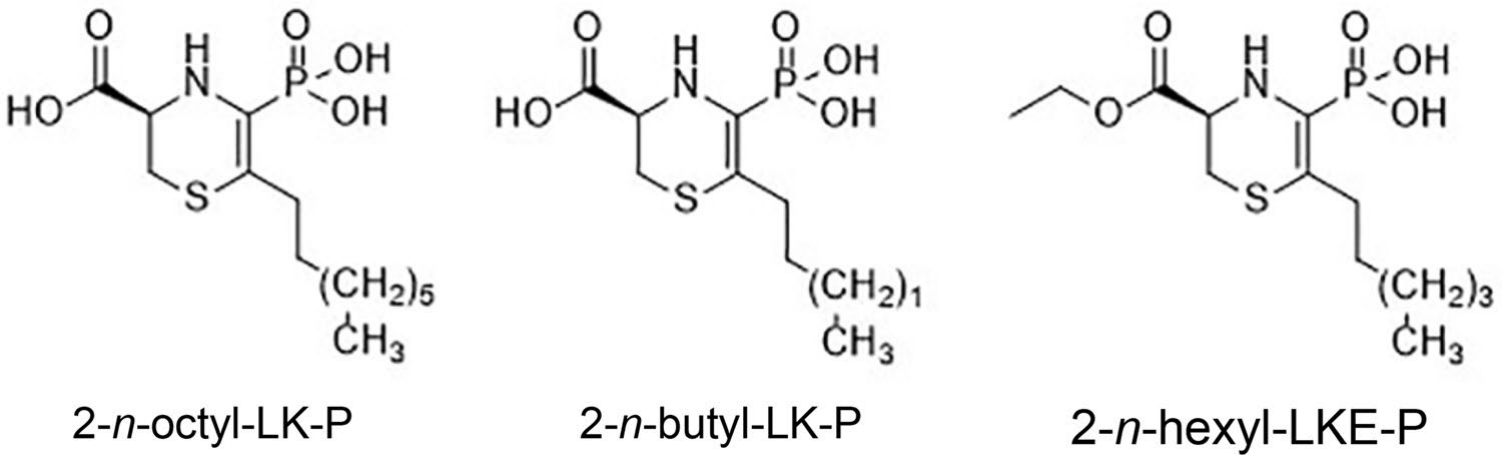
Chemical structures of lanthionine ketenamine (LK) analogs used in this study: 2-*n*-octyl-LK-P, 2-*n*-butyl-LK-P, and 2-*n*-hexyl-LKE-P.

Three phosphonate LK analogs were synthesized: 2-*n*-octyl-LK-P, 2-*n*-butyl-LK-P and 2-*n*-hexyl-LKE-P using standard Michaelis-Arbuzov (MA) reaction conditions (Fig. 1). Electrostatic interactions of the 3-carboxylate group were enhanced by increasing the charge density or the hydrogen bonding opportunities near this corner of the molecule^29^ in order to generate different LK analogs.

**Figure 2:**
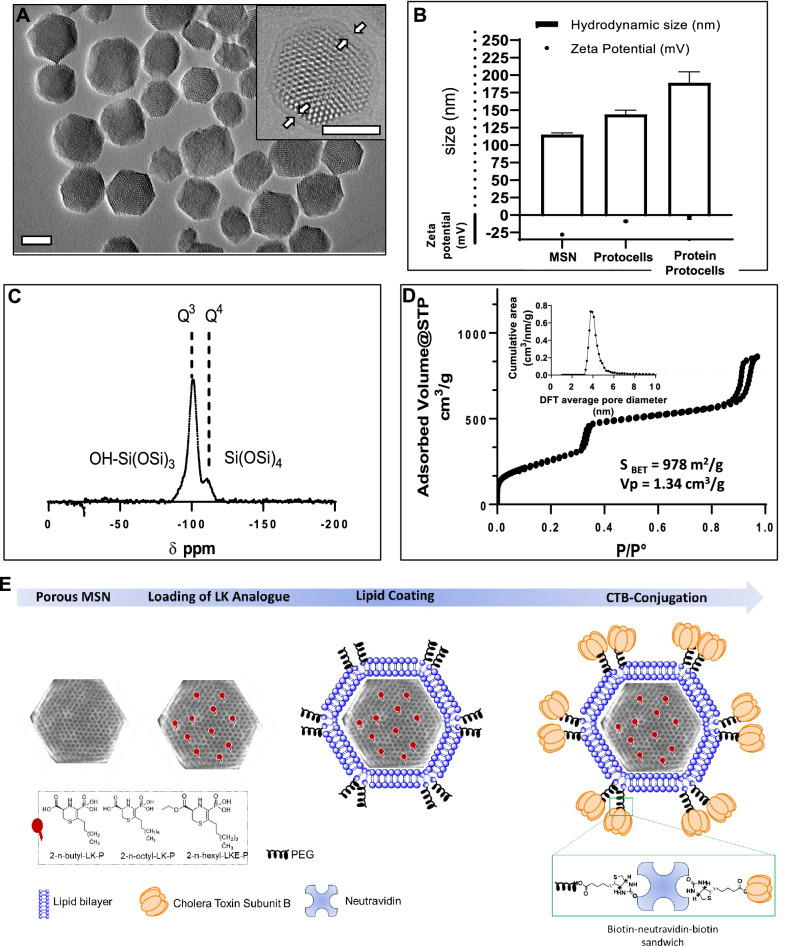
(**A**) Transmission electron microscopy image of mesoporous silica nanoparticles (MSN), inset shows a lipid-coated nanoparticle where lipid shell is indicated by white arrows. Bar, 50 nm. (**B**) Hydrodynamic diameter measured by dynamic light scattering and zeta potential. (**C**) Solid state 29Si CP-MAS NMR showing only Q4 (− 110 ppm) and Q3 (− 100 ppm) species (**D**) N2 sorption isotherm and corresponding pore volume and surface area at standard temperature and pressure (STP). (**E**) General schematic of the protocell preparation procedure. All reported values correspond to the average of at least three independent measurements.

Mesoporous Silica Nanoparticles (MSN) were generated using standard methods^34,35^. Using transmission electron microscopy a homogenous shape and hexagonal structure of MSN was visualized (Fig. 2A). Dynamic light scattering (DLS; 115 nm, PDI < 0.2) and zeta potential measurements (− 27 mV) (Fig. 2B) confirmed the colloidal homogeneity and stability of MSNs. A high degree of condensation for MSN was indicated by solid state ^29^Si CP-MAS NMR, as only Q4 (− 110 ppm) and Q3 (− 100 ppm) species (Fig. 2C) were present consistent with the step of hydrothermal treatment consolidating the MSN structure. N_2_ sorption analyses showed an isotherm type IV characteristic of MCM-41 like particles, with a pore diameter centered at 4 nm according to DFT model (Fig. 2D). The surface area (S_BET_ = 978 m^2^/g) and pore volume (Vp = 1.34 cm^3^/g) outlined the capacity of MSN to house LK analogs.

Protocell formation was confirmed by cryoelectron microscopy (Fig. 2A, inset) where the soft shell is attributed to the lipid bilayer. DLS and zeta potential also confirmed the coating by a hydrodynamic size increase (size = 149 nm, PDI < 0.2) and decrease of the net negative charge of MSN towards the neutral domain (Fig. 2B) due to the presence of PEG in the lipid shell. CTB-protocells were mainly characterized by colloidal and charge measurement (Fig. 2B) where the additional size increase and the neutral charge suggest the incorporation of the CTB moieties within the protocell lipid bilayer (Fig. 2E). The loading efficiency of CTB-protocells was estimated at ~ 50% based on absorbance measurements in the remaining supernatant. The final mass ratio was 1:2 LK:CTB-protocells (246.6 ± 44.1 μg for 2-*n*-octyl-LK-P, 262.5 ± 8.7 μg for 2-*n*-butyl-LK-P and 241.9 ± 4.3 μg for 2-*n*-hexyl-LKE-P loaded in 500 μg of MSN).

### CTB-protocells display substantial cell uptake and lack of effects on autophagy flux

CTB-protocells were internalized (evident by AlexaFluor 647 fluorescence) in 99.7% of differentiated motor neuron-like NSC-34 cells displaying mCherry and GFP fluorescence expression after transfection with the mCherry-GFP-LC3 plasmid (n = 316 out of 317 cells). CTB-protocells were identified in both the soma and dendrites of differentiated motor neurons. The mean fractional area occupied by CTB-protocells was 7.2 ± 0.4% of cell area (median: 5.7%; IQR 2.7 to 9.3), with no evidence of differences across LK analogs or vehicle loaded protocells.

The possible effect of CTB-protocell treatment on the protein levels of the microtubule-associated protein light chain 3 (LC3) in its nonlipidated (LC3-I) and lipidated (LC3-II) form and its pattern of conversion (LC3-I to LC3-II) were measured using Western blot after 24 h treatment in the presence or absence of bafilomycin. A mixed linear model was run with LK analog treatment, CTB-protocell loading, bafilomycin treatment and their interaction as fixed effects, and experimental batch as a random effect. Unloaded CTB-protocells did not change the expression of LC3-I, LC3-II, or the ratio of LC3-II to LC3-I compared to bafilomycin control (Supplementary Figs. S1 and S2), indicating no effect to induce autophagy flux. In the absence of bafilomycin, treatment with unloaded CTB-protocells did not change expression of LC3-I, LC3-II or their ratio compared to controls, indicating that the protocells also do not block autophagy flux.

### CTB-protocells loaded with 2-n-octyl-LK-P increase autophagy flux

**Figure 3:**
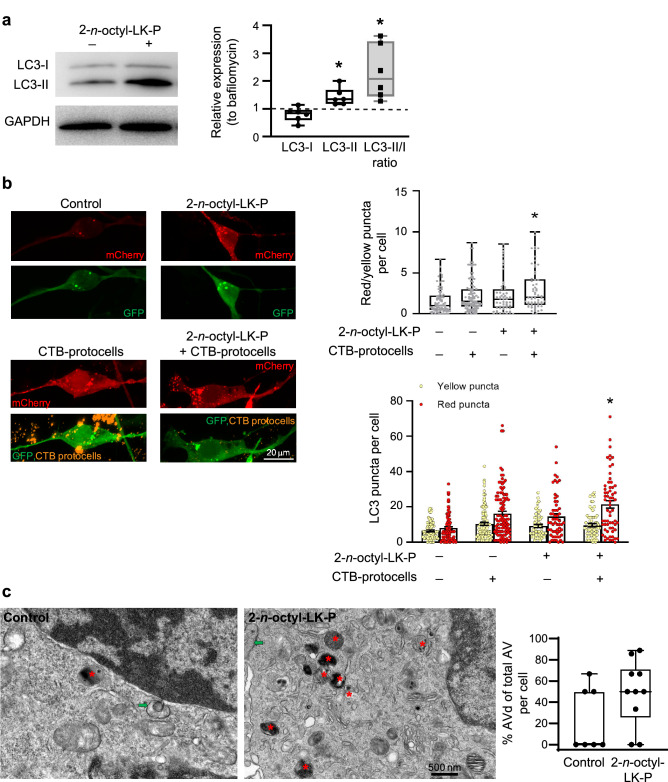
Autophagy flux in motor neurons after treatment with 2-*n*-octyl-LK-P. (**a**) Western blot analysis of LC3 protein expression in motor neurons after treatment with 2-*n*-octyl-LK-P in the presence of bafilomycin. Original uncropped blots are presented in Supplementary Fig. S3. Data are presented as a box plot, with the box extending from the 25th to 75th percentiles, the middle of the box at the median, and the whiskers at the minimum and maximum values. Each point represents protein expression from NSC-34 cells from a well of a 6-well plate. *, significantly different than bafilomycin treatment (p < 0.05); (**b**) Representative images of cells transfected with mCherry-GFP-LC3 plasmid followed by treatment with 2-*n*-octyl-LK-P or CTB-protocells containing 2-*n*-octyl-LK-P. n = 4 biological samples with a minimum of 20 cells per sample. Each point in the box plot/bar graph represents a single cell. *, significantly different than control treatment (p < 0.05); (**c**) Transmission electron microscopy images of NSC-34 cells with or without 2-*n*-octyl-LK-P treatment showing initial autophagic vacuoles (marked as green arrows) and degradative autophagic vacuole (AVd, marked as red asterisk). n = 10 cells per group from 2 biological samples. Each point in the box plot represents a single cell.

Protein analyses showed that 2-*n*-octyl-LK-P treatment in the presence of bafilomycin did not change expression of LC3-I (F_1,11_ = 4; p = 0.08) but did significantly increase LC3-II (F_1,11_ = 12; p < 0.01) compared to treatment with bafilomycin alone (Fig. 3a), indicating an increase in the synthesis of autophagy-related membranes. In the presence of bafilomycin, treatment with 2-*n*-octyl-LK-P increased the ratio of LC3-II to LC3-I compared to bafilomycin control (F_1,11_ = 11; p < 0.01).

**Figure 4:**
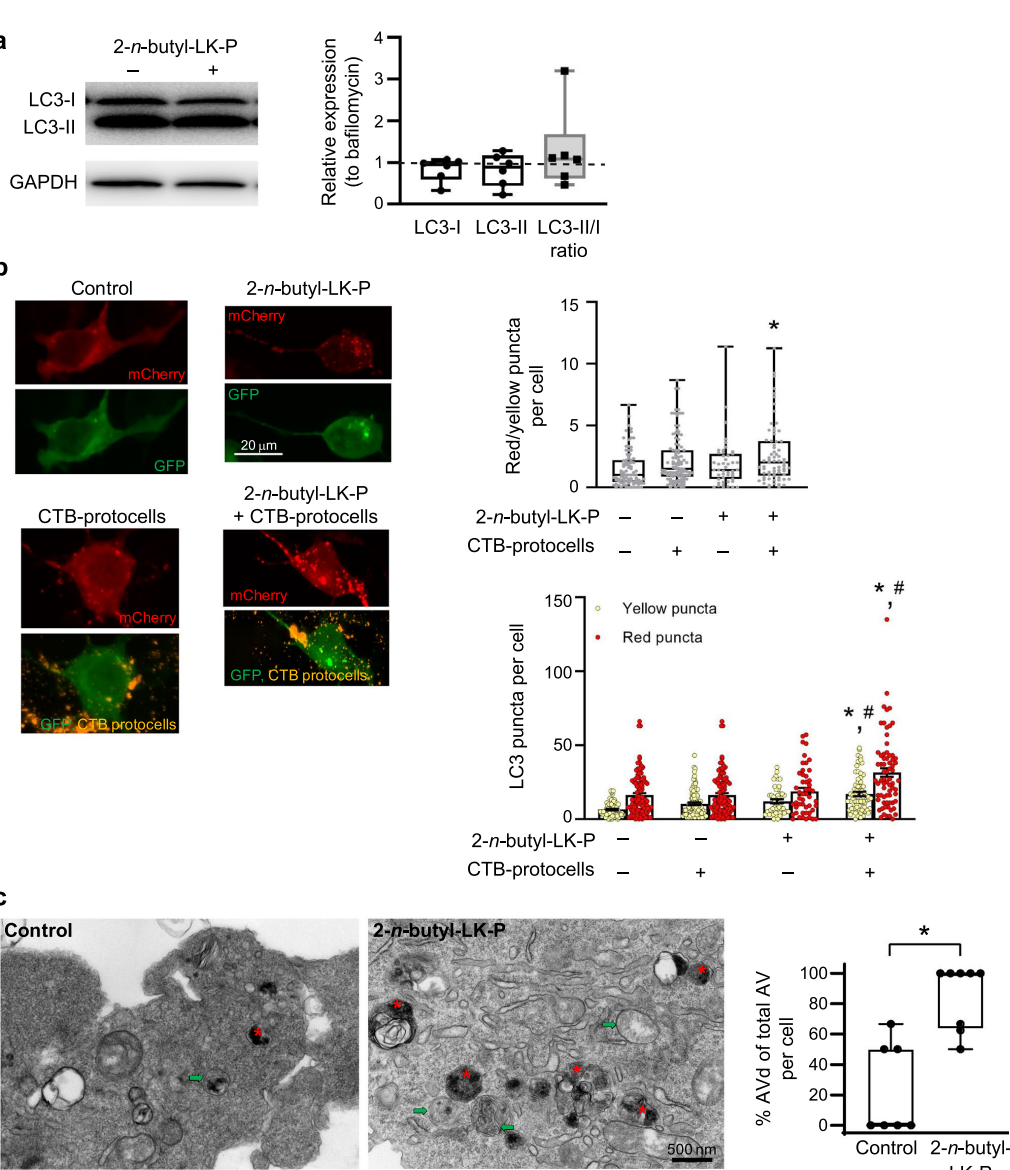
Autophagy flux in motor neurons after treatment with 2-*n*-butyl-LK-P. (**a**) Protein expression of LC3-I, LC3-II or the ratio compared to treatment with bafilomycin alone. Original uncropped blots are presented in Supplementary Fig. S4. Data are presented as a box plot, with the box extending from the 25th to 75th percentiles, the middle of the box at the median, and the whiskers at the minimum and maximum values. Each point represents protein expression from NSC-34 cells from a well of a 6-well plate; (**b**) Confocal images of NSC-34 cells transfected with mCherry-GFP-LC3 plasmid and treated with 2-*n*-butyl-LK-P or CTB-protocells containing 2-*n*-butyl-LK-P. n = 4 biological samples with a minimum of 20 cells per sample. Each point in the box plot represents a single cell. *, significantly different than control treatment (p < 0.05); ^#^, significantly different than control CTB-protocell treatment; (**c**) EM image analysis of AVd (red asterisk) over total AV per cell after 2-*n*-butyl-LK-P treatment compared to control cells. n = 8 cells per group from 2 biological samples. Each point in the box plot represents a single cell. *, significantly different (p < 0.05).

**Figure 5:**
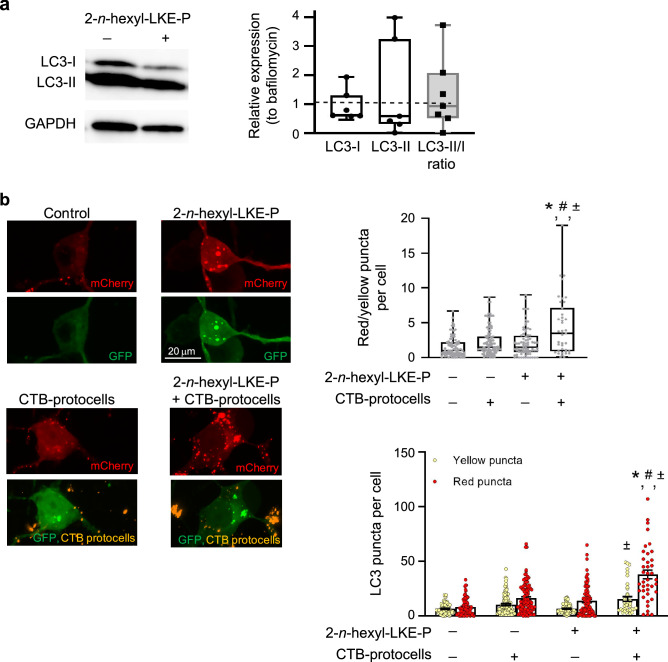
Changes of autophagy flux in motor neurons treated with 2-*n*-hexyl-LKE-P or CTB-protocells loaded with 2-*n*-hexyl-LKE-P. (**a**) Western blot analysis of LC3-I, LC3-II or the ratio after 2-*n*-hexyl-LKE-P treatment in the presence of bafilomycin compared to bafilomycin alone. Original uncropped blots are presented in Supplementary Fig. S5. Data are presented as a box plot, with the box extending from the 25th to 75th percentiles, the middle of the box at the median, and the whiskers at the minimum and maximum values. Each point represents protein expression from NSC-34 cells from a well of a 6-well plate; (**b**) Representative confocal images of NSC-34 cells transfected with mCherry-GFP-LC3 plasmid and treated with 2-*n*-hexyl-LKE-P or CTB-protocells containing 2-*n*-hexyl-LKE-P. Number of red-only puncta/cell as well as the number of yellow puncta/cell was quantified in different treatment groups. n = 5 biological samples with a minimum of 20 cells per sample. Data are presented as mean ± standard deviation of the mean. Each point represents a single cell. *, significantly different than control treatment (p < 0.05); ^#^, significantly different than control CTB-protocell treatment; ± , significantly different than 2-*n*-hexyl-LKE-P alone.

To analyze autophagy flux without the need of a lysosomal inhibitor such as bafilomycin, we used an mCherry-GFP-LC3 fluorescence plasmid. mCherry and GFP fluorescence was measured in NSC-34 cells after treatment with either vehicle, unloaded CTB-protocells, LK analog, or CTB-protocells loaded with LK analog. For all three LK analogs, autophagy flux was determined from the ratio of the number of red-only puncta (expressing mCherry) to the number of yellow puncta (expressing both GFP and mCherry) per cell. An overall mixed linear model including all 3 LK analogs was run, with LK analog treatment, CTB-protocell loading and their interaction as fixed effects and date of experiment (i.e., batch) as a random effect. There was an overall effect of CTB-protocells (F_1,294_ = 28; p < 0.01) and LK analog (F_3,226_ = 7; p < 0.01) but no interaction (F_1,482_ = 3; p = 0.05) on red/yellow puncta per cell. In addition, there was an overall effect of CTB-protocells (F_1,533_ = 35; p < 0.01) and LK analog (F_3,391_ = 10; p < 0.01) and their interaction (F_3,504_ = 10; p < 0.01) on number of red puncta per cell. There was also an overall effect of CTB-protocells (F_1,534_ = 15; p < 0.01) and LK analog (F_3,414_ = 6; p < 0.01) and their interaction (F_3,513_ = 7; p < 0.01) on number of yellow puncta per cell. The results of post-hoc Tukey–Kramer HSD tests will be presented for each LK analog separately (Figs. 3b, 4b, 5b). Importantly, treatment with unloaded CTB-protocells did not have an effect on the number of red or yellow puncta, or the ratio of the number of red to yellow puncta, suggesting that CTB-protocells alone have no effect on autophagy in differentiated NSC-34 cells.

Autophagy flux (red/yellow puncta per cell) was ~ twofold greater after treatment with CTB-protocells loaded with 2-*n*-octyl-LK-P compared to control cells (p < 0.05). The number of red puncta/cell was significantly increased after treatment with CTB-protocells loaded with 2-*n*-octyl-LK-P (p < 0.05; Fig. 3b) compared to control treatment. There was no effect of 2-*n*-octyl-LK-P or CTB-protocells loaded with 2-*n*-octyl-LK-P on yellow puncta per cell. These results suggest that 2-*n*-octyl-LK-P increased autolysosome formation and that increasing the internalization of 2-*n*-octyl-LK-P using a targeted delivery mechanism such as CTB-protocells enhanced autophagy flux in motor neurons.

EM images of NSC-34 protocells loaded with 2-*n*-octyl-LK-P were taken to further describe the autophagy flux induction. Initial autophagic vacuoles (AVi, marked as green arrows in Fig. 3c) can be identified by their content and double membrane. The degradative autophagic vacuoles (AVd, marked as red asterisk in Fig. 3c) can be identified by their partially degraded, electron-dense content. Although these results did not reach significant difference compared to control cells due to the low number of cells sampled, the percent of AVd (out of the total number of AV) was increased twofold in 2-*n*-octyl-LK-P treated cells (F_1,16_ = 3; p = 0.12).

### CTB-protocells loaded with 2-n-butyl-LK-P increase autophagy flux

Protein analysis showed that 2-*n*-butyl-LK-P treatment in the presence of bafilomycin did not change expression of LC3-I (F_1,10_ = 2; p = 0.23) or LC3-II (F_1,10_ = 1; p = 0.35) compared to treatment with bafilomycin (Fig. 4a). In addition, in the presence of bafilomycin, treatment with 2-*n*-butyl-LK-P did not change the ratio of LC3-II to LC3-I compared to bafilomycin control (F_1,10_ < 1; p = 0.54).

From confocal imaging, autophagy flux (red/yellow puncta per cell) was ~ 80% greater after treatment with CTB-protocells loaded with 2-*n*-butyl-LK-P compared to control cells (p < 0.05). The number of red puncta/cell was significantly greater after treatment with CTB-protocells loaded with 2-*n*-butyl-LK-P (p < 0.05; Fig. 4b) compared to control treatment and unloaded CTB-protocell treatment. The number of yellow puncta/cell was also significantly greater after treatment with CTB-protocells loaded with 2-*n*-butyl-LK-P (p < 0.05) compared to control treatment and unloaded CTB-protocell treatment.

With EM image analysis we observed an increase in the ratio of AVd over total AV per cell after 2-*n*-butyl-LK-P treatment compared to control cells (Fig. 4c; F_1,14_ = 21; p < 0.01).

### CTB protocells loaded with 2-n-hexyl-LKE-P increase autophagy flux

Compared to treatment with bafilomycin (Fig. 5a), 2-*n*-hexyl-LKE-P treatment in the presence of bafilomycin did not result in an overall change in the relative expression of LC3-I or LC3-II (F_1,28_ < 1; p = 0.88 and F_1,28_ < 1; p = 0.70). In the presence of bafilomycin, 2-*n*-hexyl-LKE-P treatment also did not affect the ratio of LC3-II to LC3-I (F_1,28_ < 1; p = 0.46).

Autophagy flux analysis using the mCherry-GFP-LC3 plasmid showed an effect on red/yellow puncta per cell of 2-*n*-hexyl-LKE-P when delivered using CTB protocells. Post-hoc Tukey analysis showed that autophagy flux (red/yellow puncta per cell) was ~ 3 times greater in cells treated with CTB-protocells loaded with 2-*n*-hexyl-LKE-P compared to control cells and was also significantly greater than treatment with 2-*n*-hexyl-LKE-P alone or unloaded CTB protocells (p < 0.05; Fig. 5b). The number of red-only puncta/cell was significantly greater in cells treated with CTB-protocells loaded with 2-*n*-hexyl-LKE-P compared to cells treated with 2-*n*-hexyl-LKE-P alone, CTB-protocells alone, or control cells (p < 0.05). The number of yellow puncta per cell was significantly greater in cells treated with CTB-protocells loaded with 2-*n*-hexyl-LKE-P than with 2-*n*-hexyl-LKE-P alone (p < 0.05).

## Discussion

In this study, we show that delivery of LK analogs to motor neurons via CTB-protocells induces autophagy flux in motor neuron-like cells. Modulating autophagy specifically in motor neurons using a targeted delivery system represents an innovative therapeutic approach to treat various motor neuron disorders. The use of CTB-protocells to deliver autophagy modulators to motor neurons helps overcome common obstacles encountered with traditional drugs, such as low pharmacological specificity for their target and off-target effects.

Autophagy regulation occurs through two ubiquitin-like conjugation pathways^36^. One involves the covalent binding of Atg12 to Atg5 at the phagophore level, and its dissociation after autophagosome consolidation. The other mechanism involves the conjugation of microtubule-associated protein 1 light chain 3 (LC3) to phosphatidylethanolamine (PE) to form LC3-II (lipidated LC3). Lipidated LC3 (LC3-II) binds to the elongating, double membrane phagophore and remains associated with the inner and outer-membranes of the autophagosome (before binding to the lysosome) and the autophagolysosome (even after fusion with lysosome). Static assessments of autophagy include monitoring the increase in autophagosomes, but this increase can be consequence of an increase in productive autophagic responses (increased on-rate) or a decrease in the formation or activity of autolysosomes (decreased off-rate). The gold standard for measuring autophagy activity is monitoring LC3 turnover, LC3 subcellular distribution, or its flux through the autophagy pathway^37^.

To evaluate autophagy flux in motor neurons, we used electron microscopy (EM), protein measurements of the conversion ratio between LC3-II and LC3-I in the presence of vacuolar ATP-ase inhibitor bafilomycin, and a dynamic fluorescent reporter mCherry-GFP-LC3 in a mouse motor neuron-like hybrid cell line (NSC-34). The use of EM in autophagmportant due to its ability to reveal the morphology of autophagic structures in their natural environment, at very high resolution (in the nm range). The approach of comparing LC3 lipidation in the presence of a lysosomal inhibitor such as bafilomycin helps differentiate between autophagy stimulation and late-stage blockage of autophagosome (on- vs. off-rates). By intentionally blocking LC3-II metabolism with the addition of bafilomycin, an increase in LC3-II levels reflects autophagy initiation, not decreased formation or activity of the autolysosomes. However, EM and protein measurements should not be used alone to quantify changes in autophagy^37^. The mCherry-GFP-LC3 reporter system is an efficient method to measure autophagy flux by monitoring the incorporation of the mCherry-GFP-LC3 fusion protein into autophagosomal and autophagolysosomal membranes^38,39^. Importantly, the use of this dynamic reporter allows measuring autophagy flux without the need for a lysosomal inhibitor such as bafilomycin^37,40^.

Autophagy is a viable therapeutic target for motor neuron dysfunction and diseases such as spinal cord injury^41^, amyotrophic lateral sclerosis^42^, and spinal muscular atrophy^43^. Autophagy is induced to ensure cell survival under conditions of stress such as absence of growth factors, low oxygen levels, or nutrient starvation^44^. Defects of autophagy or simply insufficient autophagy in motor neurons can produce progressive accumulation of protein aggregates and/or defective organelles that may disturb axonal trafficking and induce cell toxicity, among other effects^45^. Autophagy is pharmacologically manipulated using derivatives of the microbial macrolide rapamycin which inhibits mTORC1. mTOR inhibitors affect protein, nucleotide, and lipid synthesis as well as glutamine metabolism and glycolysis, all via autophagy-independent mechanisms. These effects confound whether any beneficial effects of mTOR inhibitors reflect increased autophagy or other effects^46^. Another specificity-related issue that most autophagy modulators have is that they do not preferentially target one cell type. Thus, evaluating the actual impact of autophagy modulators in disease scenarios in which autophagic responses may have opposite effects in different tissue compartments, is challenging. No endogenous mammalian substance has been reported that engages the mTOR pathway to produce rapamycin-like effects. Despite not knowing the exact mechanism of action, LK has been shown to significantly increase autophagy with possible downstream interactions with ULK1 or other proteins that regulate mTOR (instead of acting directly in mTOR), making it a novel candidate for a potential therapeutic^28^. LK is the cyclic ketenamine form of the mono-alpha-ketoacid form of lanthionine, which is typically produced by the transamination with the alpha-ketoacid form of methionine, alpha-keto-methylthiolbutyrate (αKMB), catalyzed by glutamine transaminase K^21,25,29^. Replacement of the 3-carboxylate with a phosphonate group and addition of alkyl groups to the 2-position of LK provide the lanthionine ketenamine (ester)-phosphonates (LK(E)-P^29^). The LK(E)-Ps are more potent autophagy stimulators than the lead compound, LK^29^. In this study, two phosphonate LKs and one phosphonate LKE were used in motor neurons: 2-*n*-octyl-LK-P, 2-*n*-butyl-LK-P and 2-*n*-hexyl-LKE-P.

In the current study, we used CTB-protocells to target delivery of LK analogs to motor neurons^30,31^. The CTB-protocell platform is a drug carrier system allowing for the direct delivery of payload to motor neurons. The unique coating of protocells with cholera toxin subunit B (CTB) engages biological retrograde pathways not easily established with untargeted delivery methods. We have previously shown that protocells can be engineered to specifically target motor neurons by modifying the lipid bilayer with CTB (CTB-protocell), the atoxic subunit of cholera toxin that binds the ganglioside GM1 present in neuronal membranes, and successfully deliver small molecule cargo^30,31^. In our previous study, after treating NSC-34 cells with CTB-protocells for 24 h, TEM images showed that CTB-protocells escape lysosomal degradation and are found in the cytosol^31^. Additionally, our previous study found no evidence of toxicity following 24 h CTB-protocell treatment in motor neuron-like NSC-34 cells, as neither overall cell death or proliferation were affected^31^. Importantly, CTB-protocell uptake and retrograde transport by peripheral axons was evident in vivo, as CTB-protocells were present in the phrenic nerve after intrapleural injection in Sprague–Dawley rats^30^. In the present study, LK analogs were successfully loaded in CTB-protocells, and NSC-34 motor neurons were treated with this drug delivery system. For each LK analog, delivery via CTB-protocells was necessary for the LK analog effect in modulating autophagy, as it was the only condition that significantly increased autophagy flux. These results suggest that the formulation of LK analogs into CTB-protocells enhances the efficacy of the drugs, and validate that LK analogs can exert a response when delivered intracellularly. In our study, 2-*n*-hexyl-LKE-P delivered via CTB-protocells was superior to CTB-protocell delivery of 2-*n*-octyl-LK-P or 2-*n*-butyl-LK-P because it resulted in: (1) a larger magnitude of autophagy flux induction (300%) compared to the other LK analogs (200% and 80%, respectively, compared to control treatment); and (2) a significantly greater autophagy flux than treatment with 2-*n*-hexyl-LKE-P alone or unloaded CTB protocells. In summary, CTB-protocells are suitable to deliver LK analogs to motor neurons (prior and current study), CTB-protocells increase LK analog bioavailability (current study) and presumably would decrease off-target effects of the LK analogs. We propose that CTB-protocells serve as a novel drug delivery system to motor neurons, providing peripheral access to the motor neuron system via retrograde pathways and thus an exciting new direction for treatment of motor neuron diseases.

CTB-protocells can increase the amount of LK analog delivered intracellularly to motor neurons by macropinocytosis^30^ which allows for the internalization of more than a single CTB-protocell. Based on the results for loading efficiency of LK analogs, motor neuron-like cells were treated with a lower concentration of LK analog as compared to cells being treated with the LK analog alone (~ 2× reduction). Evidence of increased effects of autophagy highlights more efficient delivery of LK analogs when using CTB-protocells. Future studies will aim to improve the loading efficiency of CTB-protocells and explore possible differences in release time for the different LK analogs. Importantly, when considering in vivo treatment, macropinocytosis by motor axon terminals is expected to be further activated during bouts of enhanced neuromuscular transmission^47^, thus offering potential activity-dependent delivery.

The use of CTB-protocells to deliver LK analogs not only allows us to understand how motor neurons respond to LK analog treatment, but also creates a tool to target other types of autophagy modulators for cell-specific autophagy modulation. Addressing the complexity of autophagy by using highly targeted autophagy modulators in disease models with cell-specific autophagic defects is key to the development of clinically viable strategies that rely on the modulation of autophagy. Also, CTB-protocells enable the development of a therapeutic strategy that is aimed at activating or inhibiting autophagy in specific cell types (including diseased and bystander cells) with one (or more) targeted drug candidate or candidates.

## Methods

### LK chemistry

Three analogs of LK, namely lanthionine ketenamine phosphonates (LK-P) or lanthionine ketenamine (ester) phosphonates (LKE-P) were synthesized using the standard Michaelis-Arbuzov reaction conditions as previously described^29^. Following this procedure, 2-*n*-octyl-LK-P, 2-*n*-butyl-LK-P, and 2-*n*-hexyl-LKE-P were prepared (Fig. 1). Their structures were confirmed by ^1^H, ^13^C and ^31^P NMR and liquid chromatography tandem UV spectrophotometry high-resolution mass spectrometry (LC/UV/HRMS)^29^.

### CTB-protocell preparation

#### Mesoporous silica nanoparticle (MSN) preparation

The method is adapted from literature^34,35^ and consists of creating homogeneous, monodisperse and hexagonally structured MSN with pore size centered at 4 nm. Particles were prepared by dissolving cetyl trimethylammonium bromide (CTAB; 290 mg) in 150 mL of aqueous ammonium hydroxide (0.32 M) in a 250 mL beaker. The reaction was covered with parafilm and heated to 50 °C in a silicone oil bath for 1 h with stirring at high speed (650 rpm). A tetraethyl orthosilicate (TEOS) solution was prepared at 1 M in ethanol (3 mL) to which 3-aminopropyltriethoxysilane (APTES) (3 µL, Sigma Aldrich) was added and the whole mix was added to the CTAB solution. The mixture was stirred vigorously at 50 °C for 1 h followed by overnight incubation in the 50 °C oil bath without stirring. The remaining volume was then transferred to a 100 mL glass bottle and capped for 24 h hydrothermal treatment at 70 °C in the oven in static conditions. The MSN suspension was then centrifuged at 50,000 rcf for 15 min and particles were washed with ethanol by resuspending the pellets in 10 mL (per tube) under sonication followed by another centrifugation step at 50,000 rcf for 15 min. CTAB removal was achieved by resuspending the particles in 20 mL of 6 g/L ammonium nitrate in ethanol and sonicating at 40 °C for 20 min. Particles were collected by centrifugation, resuspended in 15 mL of ethanolic HCl solution (1%) and sonicated for 15 min. This step was repeated twice and finally the particles were washed with 90% ethanol followed by 100% ethanol (each time by resuspension and centrifugation steps), collected by centrifugation, and stored suspended in ethanol. Labeling of MSN occurred by incubation of 10 mg of MSN with AlexaFluor 647-NHS (Succinimidyl Ester, (Thermofisher, 0.2 mg, 1 mg/mL in N,N-Dimethylformamide solvent) for 1 h. The MSN-AlexaFluor647 were washed by ethanol and used without further purification.

#### Formation of liposomes

Lipid films were formed by drying a lipid mix consisting of 1,2-distearoyl-sn-glycero-3-phosphocholine (DSPC, 147 µL, 25 mg/mL), cholesterol (19 µL, 25 mg/mL) and 1,2-distearoyl-sn-glycero-3-phosphoethanolamine-*N*-[amino(polyethylene glycol)-2000] (ammonium salt) (DSPE-PEG2k, 35 µL, 25 mg/mL) at a molar ratio of (75/20/5) (Avanti Polar Lipids, Birmingham, AL) using a speed evaporator (1 mbar, 5 min, 22 °C) in a scintillation vial, followed by resuspension in PBS (5 mg liposomes/1 mL PBS) in the same vial with 15 min sonication using ultrasonicator at 25 °C.

#### Loading of LK analogs and protocell formation (addition of liposomes to the MSN core)

Loading of LK analogs (500 µg) in MSN (500 µg) was achieved by soaking MSN in a solution of LK analog (0.1 M in DMSO) for 1 h. The suspension was centrifuged at 15,000 rcf and the pellet was suspended in water. Liposomes were added to the LK-loaded MSN suspension under sonication for 1 min, centrifuged and the pellet was washed with 500 µL of PBS. LK-P or LKE-P loading was quantified by measuring the absorbance (λ = 280 nm) of the combined supernatant and comparing it with a standard curve with LK(E)-P in solvent. CTB was conjugated to the protocells using the NeutrAvidin/biotin conjugation strategy as described previously^34^.

#### Characterization of MSN structure

TEM images of MSNs were acquired on a JEOL 2010 (Tokyo, Japan) instrument equipped with a Gatan Orius digital camera system (Warrendale, PA) under a 200 kV hydrodynamic size, as in previous studies^34,48^. All samples for DLS measurements were suspended in distilled water or ethanol at a 1 mg/mL concentration and washed three times through centrifugation. DLS measurements for each sample were obtained in triplicate at 25 °C and then the Z-average diameter (by intensity) was used for all reported hydrodynamic size values. Zeta potential data were acquired on a Malvern Zetasizer Nano-ZS equipped with a He–Ne laser (633 nm) and non-invasive backscatter optics. The zeta potential for all the samples was measured in distilled water in triplicates according to Smoluchowski theory.

Nitrogen adsorption–desorption isotherms of MSNs were obtained on a Micromeritics ASAP 2020 at 77 K. Samples were degassed at 60 °C for 12 h before measurements. The surface area was calculated following the Brunauer–Emmet–Teller (BET) equation and the pore size was obtained by density functional theory (DFT). Solid-state NMR was performed on Avance III Widebore 300 with a 7 mm ZrO_2_ rotor.

### Cell culture and treatment

NSC-34 motor neuron-like cells were propagated in 75 cm^2^ flasks in Dulbecco's modified eagle medium/nutrient mixture F-12 with GlutaMAX (ThermoFisher Scientific; Waltham, MA) supplemented with 10% fetal bovine serum and 2% penicillin–streptomycin. Cells were used up to passage six. Cells were differentiated for 24 h with serum-deprived media prior to any autophagy study or plasmid transfection^49^. After differentiation, the medium was exchanged for media containing 100 µg/mL of 2-*n*-octyl-LK-P, 2-*n*-butyl-LK-P, or 2-*n*-hexyl-LKE-P; or 100 μg/mL CTB-protocells containing LK-P or LKE-P analog. Cells were treated for 24 h. In some cases, 100 nM lysosomal protease inhibitor bafilomycin-A1 (bafilomycin; MilliporeSigma, St Louis, MO) was added in the last 4 h, as recommended in order to allow comparisons of the amount of LC3 that is lysosomally degraded^37^.

### Protein measurements

At termination of treatment with LK analog (without CTB-protocells), the cell culture medium was removed, cells were washed with PBS (MilliporeSigma), trypsinized and centrifuged to remove supernatant and collect the cell pellet. Cells were lysed on ice in Pierce radioimmunoprecipitation (RIPA) buffer containing protease and phosphatase inhibitors (ThermoFisher Scientific). Protein concentration was determined using the Bio-Rad DC protein assay (Bio-Rad Laboratories, Hercules, CA). Samples were diluted 1:1 in Laemmli buffer (Bio-Rad), electrophoretically separated under denaturing conditions on 10% SDS-PAGE Criterion gels (Bio-Rad) and transferred to polyvinylidene difluoride membranes (Bio-Rad). Membranes were blocked in tris-buffered saline (TBS; pH 7.5) with 5% BSA and 0.1% tween-20 (MilliporeSigma), followed by an overnight incubation with primary antibody for LC3B (Cell Signaling, Danvers, MA; rabbit polyclonal, #3868; RRID:AB_2137707 (1:1000) or GAPDH (MilliporeSigma; rabbit polyclonal, #G9545; RRID:AB_796208 (0.2 mg/mL) diluted in TBS containing 5% BSA and 0.1% tween-20. Membranes were incubated with appropriate horseradish peroxidase-conjugated secondary antibodies (Santa Cruz Biotechnology, Santa Cruz, CA), and immunodetection was performed using enhanced chemiluminescence (Pierce Biotechnology, Rockford, IL). Images were obtained and quantified with Kodak MM4000 Image Station software (Kodak Molecular Imaging Systems, New Haven, CT).

To validate the effect of bafilomycin on LC3 protein expression, a subset of experiments did not incubate differentiated NSC-34 cells with bafilomycin. As expected, bafilomycin treatment had an effect on LC3-II protein levels as well as on the ratio of LC3-II/LC3-I compared to no bafilomycin (p < 0.01). LC3-II protein expression was negligible without the use of bafilomycin (Supplementary Fig. S1).

### Dynamic fluorescent reporter measurements

#### Plasmid transfection

NSC-34 motor neuron-like cells were seeded onto 35 mm diameter dishes with 10 mm glass bottoms, previously coated with collagen. NSC-34 motor neuron-like cells were transfected with the mCherry-GFP-LC3 plasmid: pBABE-puro mCherry-EGFP-LC3B (a gift from Jayanta Debnath; Addgene plasmid # 22418; http://n2t.net/addgene:22418; RRID:Addgene_22418)^50^ using lipofectamine™ 2000 (Invitrogen) at a concentration of 10 μL/mL in the final transfection volume, according to manufacturer's protocol. Cells were incubated with the transfection mix in DMEM/F12 media without serum or antibiotics. DMEM containing 10% FBS was added to the transfection medium 6 h after initiation of transfection. After an additional 48 h, the medium was replaced with medium containing either bafilomycin, 2-*n*-octyl-LK-P, 2-*n*-butyl-LK-P, 2-*n*-hexyl-LKE-P, CTB-protocells loaded with the different LK analogs, or unloaded CTB-protocells (control). Cells were incubated at 37 °C, 5% CO_2_ with treatment for 24 h. Immediately after treatment, cells were washed with PBS and fixed with excess 4% paraformaldehyde for 15 min.

#### Confocal imaging

Cells were imaged using an inverted confocal microscope with 488 nm and 561 nm lasers and a 60× (NA 1.4) oil-immersion objective (Nikon Instruments Inc., Melville, NY). GFP (green) puncta and mCherry (red) puncta, were imaged sequentially in two channels. Confocal image stacks (0.5 µm step size) were obtained for individual cells. Inclusion criteria included cells that were separate from other cells, so that quantification of red (mCherry) or yellow (both mCherry and GFP) puncta could be performed on an individual cell basis.

#### Image processing and analysis

Confocal image stacks were processed and analyzed using NIS-Elements software (Nikon Instruments Inc.). Each image (cell) underwent blind deconvolution (point scan confocal algorithm, 5 iterations) to improve the signal/noise ratio of individual puncta and a maximum intensity projection image was made. Regions of interest (ROIs) were drawn around transfected cells, and the GFP and mCherry channels were then thresholded. An intersection layer was also created to characterize mCherry puncta compared to yellow puncta (expressing both GFP and mCherry, which corresponds to autophagosomes). On maximum intensity projection images, the number of puncta expressing only mCherry (red), or both mCherry and GFP (yellow) were measured. Autophagy flux determination was obtained by the ratio of the number of red-only puncta to the number of yellow puncta per cell, as suggested in prior work using this mCherry-GFP-LC3 plasmid^38,39^.

For initial validation of the mCherry-GFP-LC3 plasmid, one batch of cells was incubated with bafilomycin, an inhibitor of autophagosome-lysosome fusion. The number of puncta expressing only mCherry (red), or both mCherry and GFP (yellow) were measured as described above. Bafilomycin significantly decreased the ratio of the number of red-only puncta to the number of yellow puncta per cell (autophagy flux; F_1,31_ = 30; p < 0.01). As expected, bafilomycin allowed the GFP signal to persist, and there was a significant (92%) decrease in red-only puncta, per cell, compared to control cells (F_1,31_ = 20; p < 0.01).

### Electron microscopy

NSC-34 motor neuron-like cells were cultured in DMEM/F12 medium at 37 °C, 5% CO_2_ in a 35 mm dish for 24 h prior to the experiment. After motor neuron differentiation (achieved with serum deprived media), cells were incubated with LK analog (without CTB-protocells) for 4 h, washed with PBS and fixed in Trump’s fixative (1% glutaraldehyde and 4% formaldehyde in 0.1 M phosphate buffer, pH 7.2) for 24 h. Cells were then rinsed three times for 30 min with 0.1 M phosphate buffer (pH 7.2), followed by a 30 min postfix in phosphate-buffered 1% osmium tetroxide (OsO_4_). After rinsing three times with distilled water for 30 min, cells were stained with 2% uranyl acetate for 15 min at 60 °C. The cells were then rinsed in distilled water, dehydrated in progressively higher concentrations of ethanol followed by 100% propyleneoxide and embedded in Spurr’s resin. Thin (100 nm) sections were obtained using Leica EM UC7 ultramicrotome (Buffalo Grove, IL), then placed on 200 mesh copper grids and stained with lead citrate. Micrographs were acquired on a JEOL JEM-1400 transmission electron microscope (Tokyo, Japan) operating at 80 kV.

#### EM image analysis

To assess alterations in autophagy, the ratio of autophagosomes to autolysosomes was quantified in EM images of cells treated with different LK analogs^37^. Autophagosomes, also referred to as initial autophagic vacuoles (AVi), were identified based on presence of a double membrane. Late/degradative autophagic vacuoles/autolysosomes (AVd) were selected as vacuoles with one limiting membrane that contain electron dense cytoplasmic material and/or organelles at various stages of degradation.

### Statistics

All statistical evaluations were performed using standard statistical software (JMP 14, SAS Institute Inc., Cary, NC; RRID:SCR_014242). The normality of the data was tested using the Shapiro–Wilk test. Outliers were determined as being more than 2 standard deviations from the mean and no outliers were detected. Western blot and EM results were examined using one-way analysis of variance, followed by the Tukey–Kramer HSD test, when appropriate. Western blot data evaluating the effect of bafilomycin, LK analog treatment, and CTB-protocell loading (Supplementary Fig. S1) was analyzed using a mixed linear model with LK analog treatment, CTB-protocell loading, bafilomycin treatment and their interaction as fixed effects, and experimental batch as a random effect. Similarly, autophagy flux data using mCherry-GFP-LC3 reporter was examined using a mixed linear model with LK analog treatment, CTB-protocell loading and their interaction as fixed effects, and experimental batch as a random effect. It was decided a priori to perform post-hoc Tukey–Kramer HSD test on the interaction effect. Statistical significance was established at the p < 0.05 level. All experimental data are presented as mean ± SE, unless otherwise specified.

## Supplementary Information

Supplementary Figures.

## Data Availability

The datasets generated and/or analyzed during the current study are included in its supplementary information files or available from the corresponding author on reasonable request.

## References

[1] Levine B, Kroemer G (2008). Autophagy in the pathogenesis of disease. Cell.

[2] Anding AL, Baehrecke EH (2017). Cleaning house: Selective autophagy of organelles. Dev. Cell.

[3] Yang Z, Klionsky DJ (2010). Eaten alive: A history of macroautophagy. Nat. Cell Biol..

[4] Nixon RA (2013). The role of autophagy in neurodegenerative disease. Nat. Med..

[5] Farre JC, Subramani S (2016). Mechanistic insights into selective autophagy pathways: Lessons from yeast. Nat. Rev. Mol. Cell Biol..

[6] Gordon PB, Seglen PO (1988). Prelysosomal convergence of autophagic and endocytic pathways. Biochem Biophys. Res. Commun..

[7] Stromhaug PE, Berg TO, Fengsrud M, Seglen PO (1998). Purification and characterization of autophagosomes from rat hepatocytes. Biochem. J..

[8] Nikoletopoulou V, Papandreou ME, Tavernarakis N (2015). Autophagy in the physiology and pathology of the central nervous system. Cell Death Differ..

[9] Boya P, Reggiori F, Codogno P (2013). Emerging regulation and functions of autophagy. Nat. Cell Biol..

[10] Komatsu M (2007). Essential role for autophagy protein Atg7 in the maintenance of axonal homeostasis and the prevention of axonal degeneration. Proc. Natl. Acad. Sci. USA.

[11] Komatsu M (2006). Loss of autophagy in the central nervous system causes neurodegeneration in mice. Nature.

[12] Hara T (2006). Suppression of basal autophagy in neural cells causes neurodegenerative disease in mice. Nature.

[13] Carnio S (2014). Autophagy impairment in muscle induces neuromuscular junction degeneration and precocious aging. Cell Rep..

[14] Nikoletopoulou V, Sidiropoulou K, Kallergi E, Dalezios Y, Tavernarakis N (2017). Modulation of autophagy by BDNF underlies synaptic plasticity. Cell Metab..

[15] Chen A, Xiong LJ, Tong Y, Mao M (2013). Neuroprotective effect of brain-derived neurotrophic factor mediated by autophagy through the PI3K/Akt/mTOR pathway. Mol. Med. Rep..

[16] Song JW (2008). Lysosomal activity associated with developmental axon pruning. J. Neurosci..

[17] Gonzalez Porras MA, Sieck GC, Mantilla CB (2018). Impaired autophagy in motor neurons: A final common mechanism of injury and death. Physiology.

[18] Chen S, Zhang X, Song L, Le W (2012). Autophagy dysregulation in amyotrophic lateral sclerosis. Brain Pathol..

[19] Tan CC (2014). Autophagy in aging and neurodegenerative diseases: Implications for pathogenesis and therapy. Neurobiol. Aging.

[20] Chung CH (2007). Identification of lanthionine synthase C-like protein-1 as a prominent glutathione binding protein expressed in the mammalian central nervous system. Biochemistry.

[21] Hensley K, Denton TT (2015). Alternative functions of the brain transsulfuration pathway represent an underappreciated aspect of brain redox biochemistry with significant potential for therapeutic engagement. Free Radic. Biol. Med..

[22] Hensley K (2013). A derivative of the brain metabolite lanthionine ketimine improves cognition and diminishes pathology in the 3 x Tg-AD mouse model of Alzheimer disease. J. Neuropathol. Exp. Neurol..

[23] Hensley K (2010). Proteomic identification of binding partners for the brain metabolite lanthionine ketimine (LK) and documentation of LK effects on microglia and motoneuron cell cultures. J. Neurosci..

[24] Zhang W (2009). Structure of human lanthionine synthetase C-like protein 1 and its interaction with Eps8 and glutathione. Genes Dev..

[25] Shen D, Hensley K, Denton TT (2020). An overview of sulfur-containing compounds originating from natural metabolites: Lanthionine ketimine and its analogues. Anal. Biochem..

[26] Togashi K (2020). Lanthionine ketimine ester improves outcome in an MPTP-induced mouse model of Parkinson's disease via suppressions of CRMP2 phosphorylation and microglial activation. J Neurol Sci.

[27] Hensley K (2010). Emerging biological importance of central nervous system lanthionines. Molecules.

[28] Harris-White ME (2015). A cell-penetrating ester of the neural metabolite lanthionine ketimine stimulates autophagy through the mTORC1 pathway: Evidence for a mechanism of action with pharmacological implications for neurodegenerative pathologies. Neurobiol. Dis..

[29] Shen D, Hensley K, Denton TT (2018). Multiple-step, one-pot synthesis of 2-substituted-3-phosphono-1-thia-4-aza-2-cyclohexene-5-carboxylates and their corresponding ethyl esters. Bioorg. Med. Chem. Lett..

[30] Gonzalez Porras MA (2018). Uptake and intracellular fate of cholera toxin subunit b-modified mesoporous silica nanoparticle-supported lipid bilayers (aka protocells) in motoneurons. Nanomedicine.

[31] Gonzalez Porras MA (2016). A novel approach for targeted delivery to motoneurons using cholera toxin-B modified protocells. J. Neurosci. Methods.

[32] Butler KS (2016). Protocells: Modular mesoporous silica nanoparticle-supported lipid bilayers for drug delivery. Small.

[33] Sheikh KA, Deerinck TJ, Ellisman MH, Griffin JW (1999). The distribution of ganglioside-like moieties in peripheral nerves. Brain.

[34] Durfee PN (2016). Mesoporous silica nanoparticle-supported lipid bilayers (protocells) for active targeting and delivery to individual leukemia cells. ACS Nano.

[35] LaBauve AE (2018). Lipid-coated mesoporous silica nanoparticles for the delivery of the ML336 antiviral to inhibit encephalitic alphavirus infection. Sci. Rep..

[36] Feng Y, Yao Z, Klionsky DJ (2015). How to control self-digestion: Transcriptional, post-transcriptional, and post-translational regulation of autophagy. Trends Cell Biol..

[37] Klionsky DJ (2021). Guidelines for the use and interpretation of assays for monitoring autophagy (4th edition). Autophagy.

[38] Castillo K (2013). Measurement of autophagy flux in the nervous system in vivo. Cell Death Dis..

[39] Castillo K, Valenzuela V, Onate M, Hetz C (2017). A Molecular reporter for monitoring autophagic flux in nervous system in vivo. Methods Enzymol..

[40] Ueno T, Komatsu M (2020). Monitoring autophagy flux and activity: Principles and applications. BioEssays.

[41] Wang ZY, Liu WG, Muharram A, Wu ZY, Lin JH (2014). Neuroprotective effects of autophagy induced by rapamycin in rat acute spinal cord injury model. NeuroImmunoModulation.

[42] Galluzzi L, Bravo-San Pedro JM, Levine B, Green DR, Kroemer G (2017). Pharmacological modulation of autophagy: Therapeutic potential and persisting obstacles. Nat. Rev. Drug Discov..

[43] Periyakaruppiah A (2016). Autophagy modulators regulate survival motor neuron protein stability in motoneurons. Exp. Neurol..

[44] Bar-Yosef T, Damri O, Agam G (2019). Dual role of autophagy in diseases of the central nervous system. Front. Cell Neurosci..

[45] Nixon RA, Yang DS, Lee JH (2008). Neurodegenerative lysosomal disorders: A continuum from development to late age. Autophagy.

[46] Li J, Kim SG, Blenis J (2014). Rapamycin: One drug, many effects. Cell Metab..

[47] Gonzalez Porras MA, Fogarty MJ, Gransee HM, Sieck GC, Mantilla CB (2019). Frequency-dependent lipid raft uptake at rat diaphragm muscle axon terminals. Muscle Nerve..

[48] Noureddine A (2020). Engineering of monosized lipid-coated mesoporous silica nanoparticles for CRISPR delivery. Acta Biomater..

[49] Maier O (2013). Differentiated NSC-34 motoneuron-like cells as experimental model for cholinergic neurodegeneration. Neurochem. Int..

[50] N'Diaye EN (2009). PLIC proteins or ubiquilins regulate autophagy-dependent cell survival during nutrient starvation. EMBO Rep..

