# Supplementary material for: CTB-targeted protocells enhance ability of lanthionine ketenamine analogs to induce autophagy in motor neuron-like cells
Source: Sci Rep. 2023 Feb 13;13:2581. doi: 10.1038/s41598-023-29437-8 (PMC9925763; doi:10.1038/s41598-023-29437-8)
Supplement: Supplementary file 1 — Supplementary Figures. [file 41598_2023_29437_MOESM1_ESM.pdf]

CTB-targeted Protocells Enhance Ability of Lanthionine Ketenamine Analogs to Induce Autophagy in Motor Neuron-like Cells

M.A. Gonzalez Porras, H.M. Gransee, T.T. Denton, D. Shen, K.L. Webb, C.J. Brinker, A. Noureddine, G.C. Sieck and C.B. Mantilla

Supplementary Figure S1

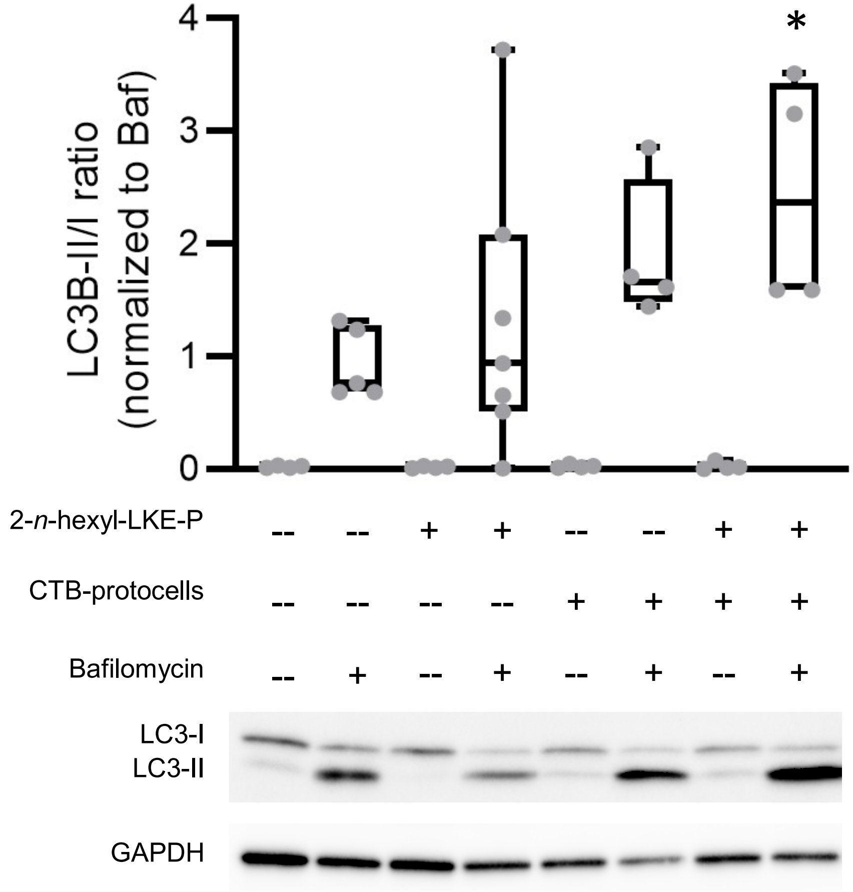

Figure S1. Western blot analysis of LC3-I, LC3-II or the ratio after treatment across all conditions (± 2-*n*-hexyl-LKE-P, ± CTB-protocells, ± bafilomycin). The no bafilomycin group had very low LC3-II/LC3-I ratios. Treatment with 2-*n*-hexyl-LKE-P via CTB-protocells show an increase in LC3-II/LC3-I ratio compared to control. Data are presented as a box plot, with the box extending from the 25th to 75th percentiles, the middle of the box at the median, and the whiskers at the minimum and maximum values. Each point represents protein expression from NSC-34 cells from a well of a 6-well plate. *, significantly different than control.

Supplementary Figure S2

Uncropped blots from Figure S1

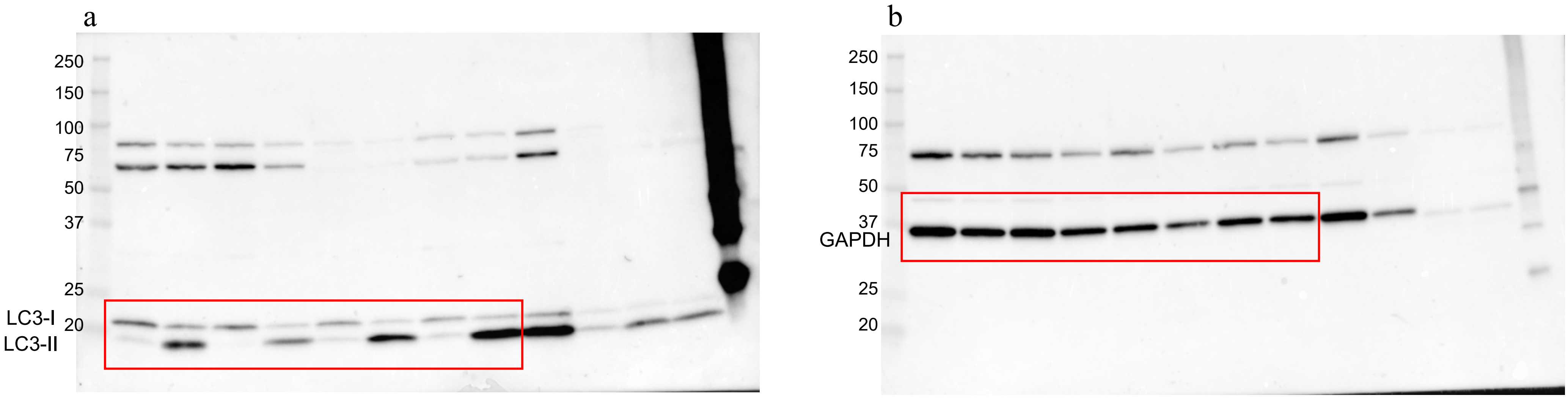

Figure S2. Original uncropped blots from Figure S1 for (a) LC3, and (b) GAPDH in NSC-34 cells after treatment across all conditions (± 2-*n*-hexyl-LKE-P, ± CTB-protocells, ± bafilomycin). Both antibodies were probed on the same blot in the following order: LC3, GAPDH. Red boxes indicate regions that were cropped and presented in Figure S1.

Supplementary Figure S3

Uncropped blots from Figure 3A

a

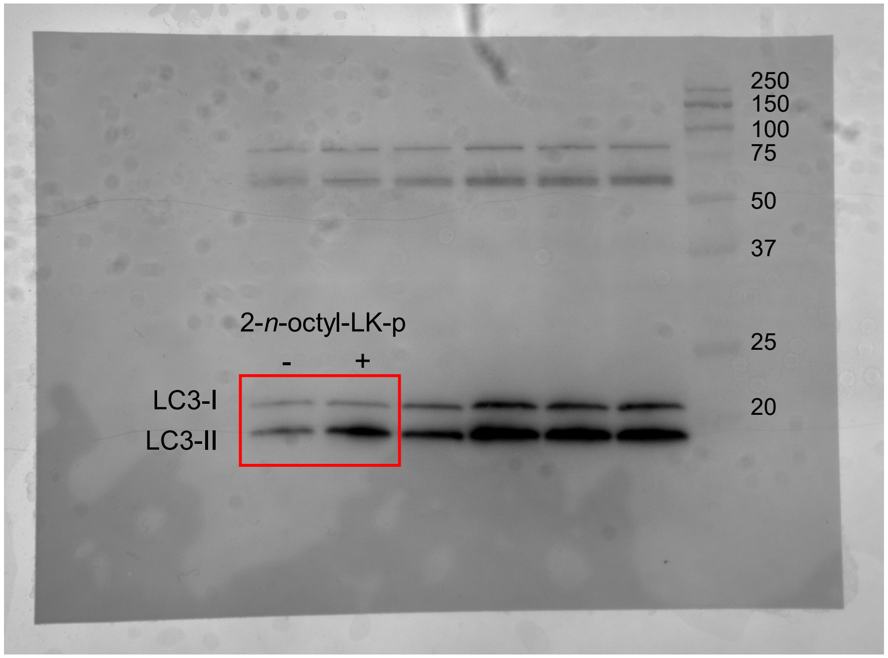

b

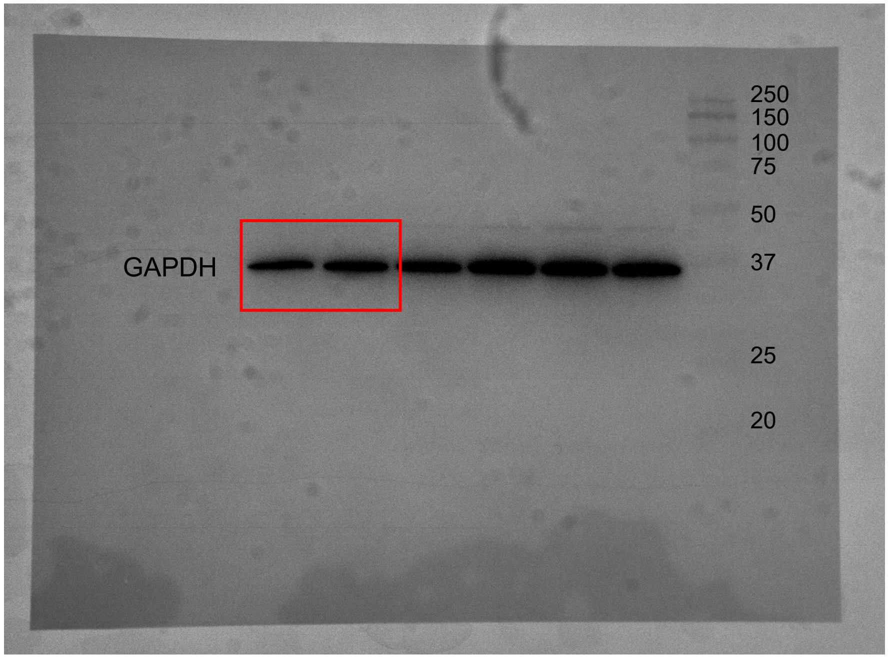

Figure S3. Original uncropped blots from Figure 3A for (a) LC3, and (b) GAPDH in NSC-34 cells treated with (+) or without (-) 2-*n*-octyl-LK-P in the presence of bafilomycin. Both antibodies were probed on the same blot in the following order: LC3, GAPDH. Red boxes indicate regions that were cropped and presented in figure within the manuscript.

Supplementary Figure S4

Uncropped blots from Figure 4A

a

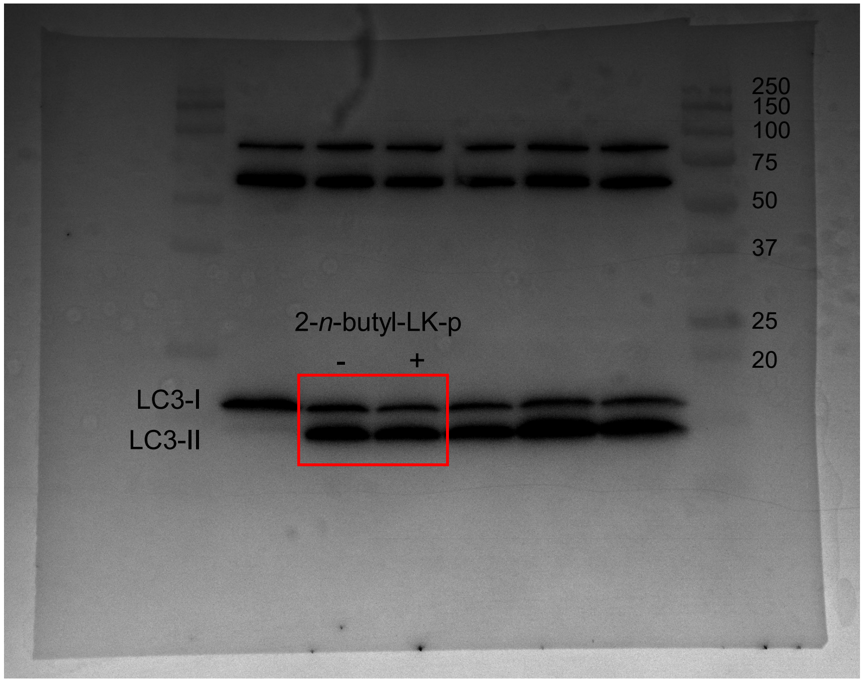

b

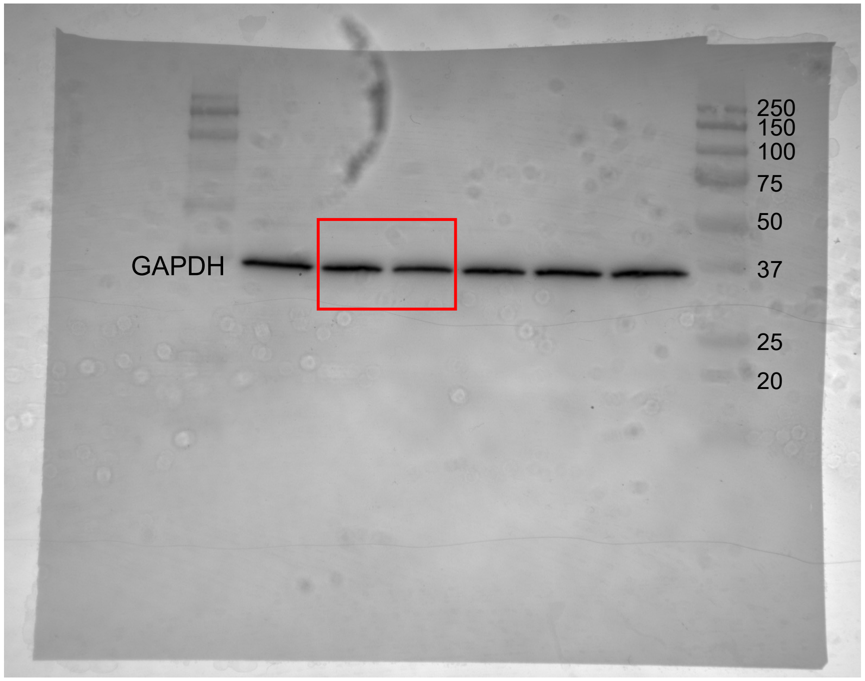

Figure S4. Original uncropped blots from Figure 4A for (a) LC3, and (b) GAPDH in NSC-34 cells treated with (+) or without (-) 2-*n*-butyl-LK-P in the presence of bafilomycin. Both antibodies were probed on the same blot in the following order: LC3, GAPDH. Red boxes indicate regions that were cropped and presented in figure within the manuscript.

Supplementary Figure S5
Uncropped blots from Figure 5A

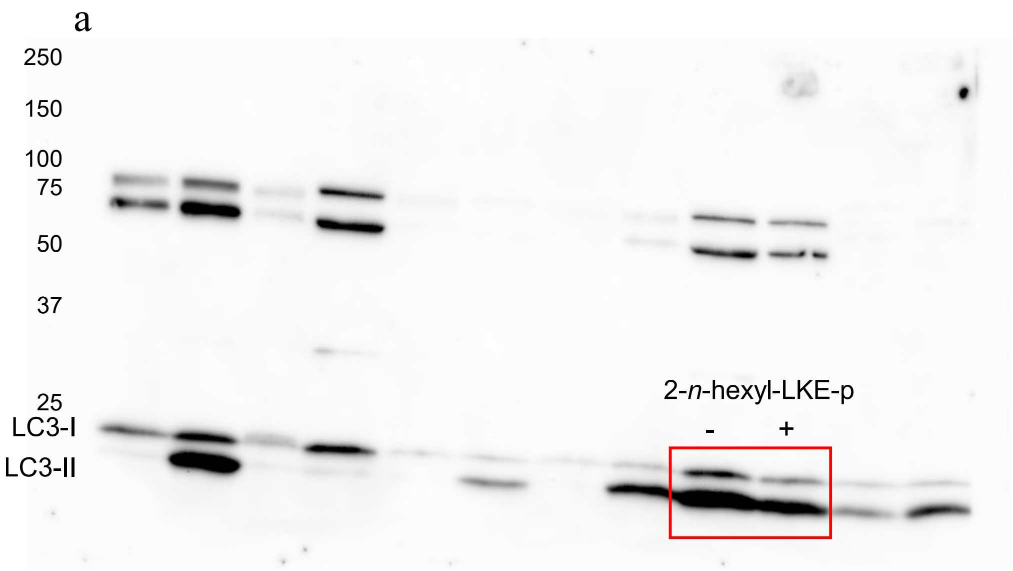

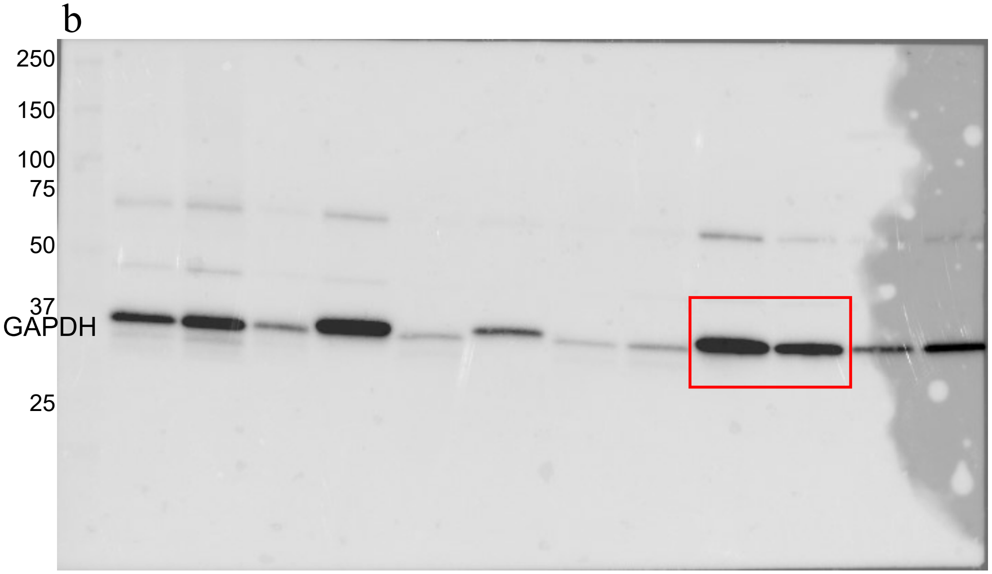

Figure S5. Original uncropped blots from Figure 5A for (a) LC3, and (b) GAPDH in NSC-34 cells treated with (+) or without (-) 2-*n*-hexyl-LKE-P in the presence of bafilomycin. Both antibodies were probed on the same blot in the following order: LC3, GAPDH. Red boxes indicate regions that were cropped and presented in figure within the manuscript.
